# Pleomorphic Adenoma in a Posterior Cervical Soft Tissue Mass: A Diagnostic Conundrum

**DOI:** 10.7759/cureus.58839

**Published:** 2024-04-23

**Authors:** Muhammad Adzha Musa, Michael Sze Liang Wong, Khairil Afif Mahmud, Mohd Aizat Abdul Aziz, Mohd Razif Mohamad Yunus

**Affiliations:** 1 Department of Otorhinolaryngology-Head and Neck Surgery, Hospital Canselor Tuanku Muhriz UKM, Kuala Lumpur, MYS; 2 Department of Otorhinolaryngology, Hospital Queen Elizabeth, Kota Kinabalu, MYS; 3 Department of Pathology, Hospital Queen Elizabeth, Kota Kinabalu, MYS

**Keywords:** head and neck tumors, posterior cervical triangle, soft tissue tumour, salivary gland tumor, pleomorphic adenoma

## Abstract

Diagnosing a soft tissue tumor in the head and neck region can be challenging due to its complex anatomy and diverse histological spectrum. This case report highlights the case of a woman who presented with a painless neck lump in the posterior triangle of the neck. Various pathological and imaging studies were suggestive of pleomorphic adenoma, which arises from the left prevertebral space. The patient underwent complete surgical excision via the transcervical approach. Pleomorphic adenoma in the posterior triangle of the neck is extremely rare and causes a diagnostic dilemma in managing soft tissue tumors of the neck.

## Introduction

The posterior neck mainly consists of soft tissue and tumors arising from this region involve a wide spectrum of differentiation. The tumors commonly originate from adipose tissue, muscle, and blood vessels but rarely from salivary glands [1,2]. The presence of heterotopic salivary glands in the posterior neck is exceptionally uncommon and tumors originating from this mesenchymal pose a diagnostic dilemma. We describe a rare case of a posterior neck soft tissue mass in which the initial cytological diagnosis revealed pleomorphic adenoma. The subsequent investigations and management of this case are described in this article.

## Case presentation

**Figure 1:**
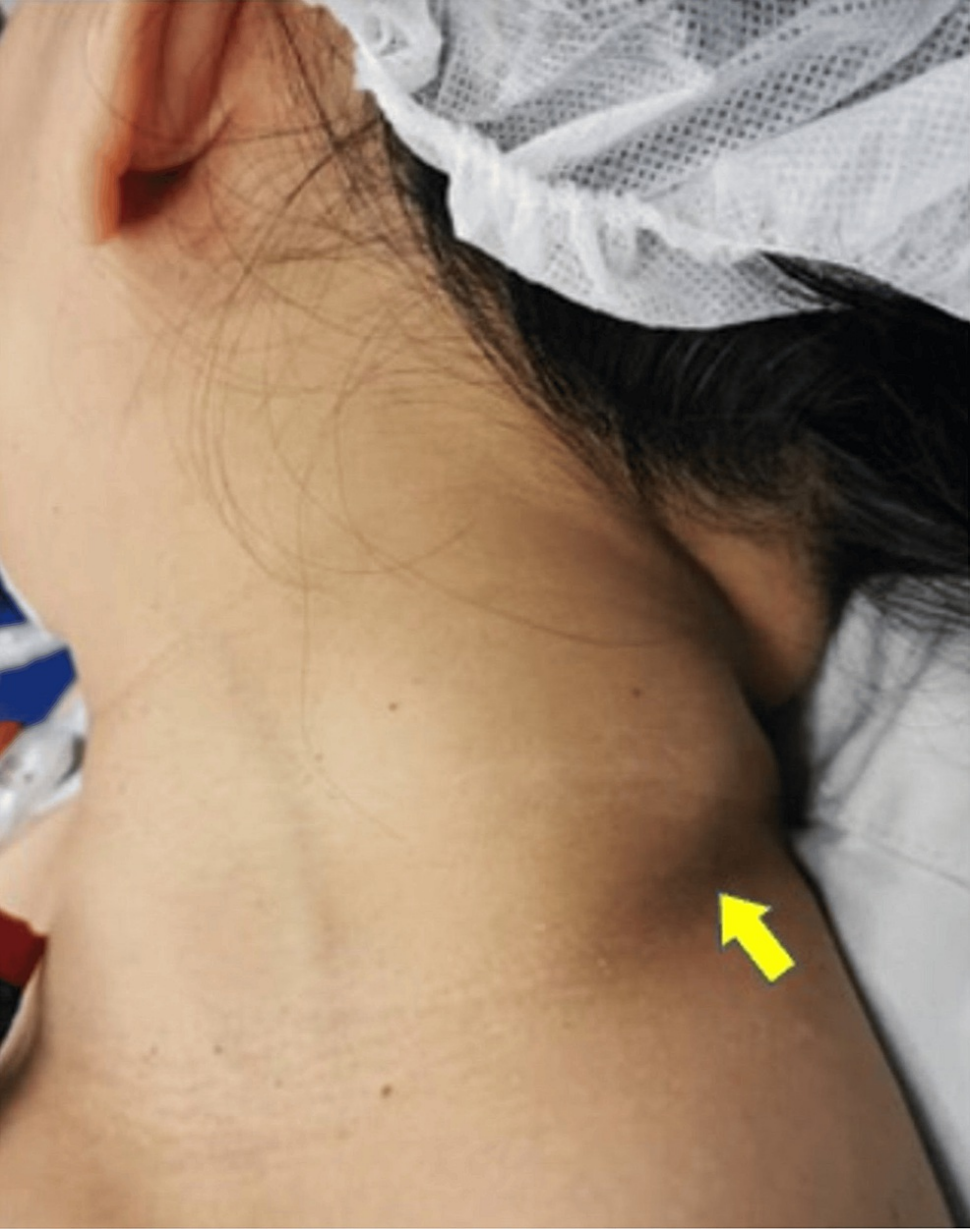
Clinical image showing swelling in the left posterior triangle of the neck measuring 5 x 4 cm (yellow arrowhead)

A 44-year-old woman presented with a painless mass on the left side of her neck for five years, which was gradually increasing in size. She denied other symptoms suggestive of upper aerodigestive tract malignancy, tuberculosis, or constitutional symptoms. Clinical examination revealed a well-defined neck swelling on the left side of the posterior triangle of the neck, measuring 5 x 4 centimeters (cm). The mass was firm, mobile, and non-tender with a smooth surface. There were no overlying skin changes (Figure 1). Other lymph nodes were not palpable. Otherwise, the ear, nose, and throat examinations were not remarkable.

**Figure 2:**
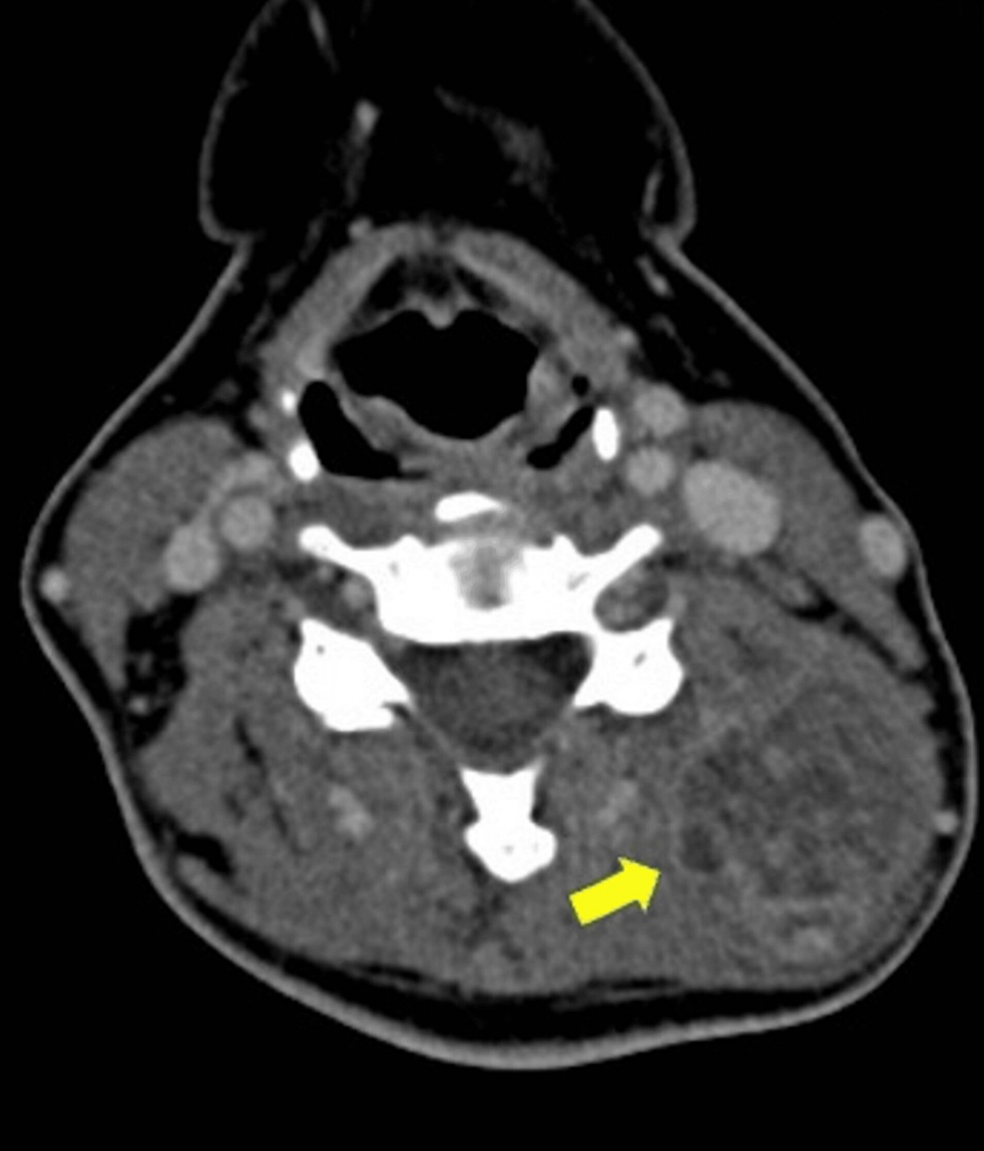
Contrasted CT scan of the neck showing a well-defined, heterogeneous, enhancing lobulated mass in the left posterior triangle of the neck measuring 3.6 x 3.2 x 4.6 cm (yellow arrowhead)

**Figure 3:**
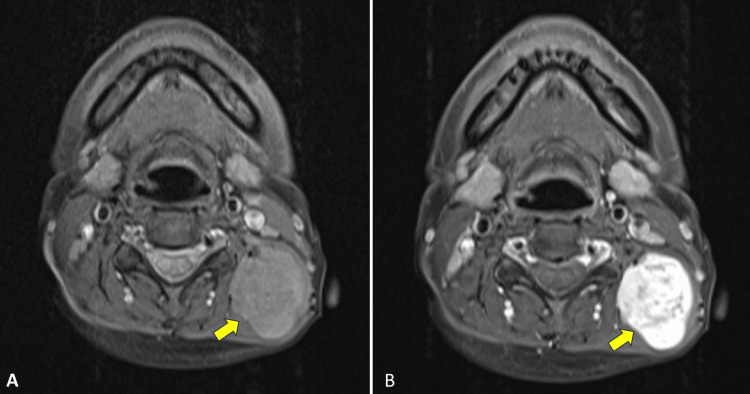
(A) The T1 MR image showing a lobulated mass with intermediate signal intensity within the left prevertebral space measuring 3.5 x 3.3 x 4.4 cm (yellow arrowhead), and (B) the mass shows enhancement after gadolinium contrast administration (yellow arrowhead)

A contrasted computed tomography (CT) scan of the neck revealed a well-defined heterogenous enhancing mass in the left posterior triangle of the neck measuring 3.6 x 3.2 x 4.6 cm between the left erector spinae muscle and the left levator scapulae muscle with no clear plane of demarcation between them (Figure 2). Magnetic resonance imaging (MRI) of the neck showed a lobulated enhancing mass within the paraspinal part of the left prevertebral space measuring 3.5 x 3.3 x 4.4 cm that appeared to arise within the intramuscular region. The mass demonstrated variable signal intensities in both T1 and T2 weighted sequences (Figure 3). Fine-needle aspiration for cytology was performed on the mass and the result showed features suggestive of pleomorphic adenoma. As the diagnosis was suspicious for a posterior neck region, a core needle biopsy was performed and the histopathological examination was consistent with pleomorphic adenoma.

**Figure 4:**
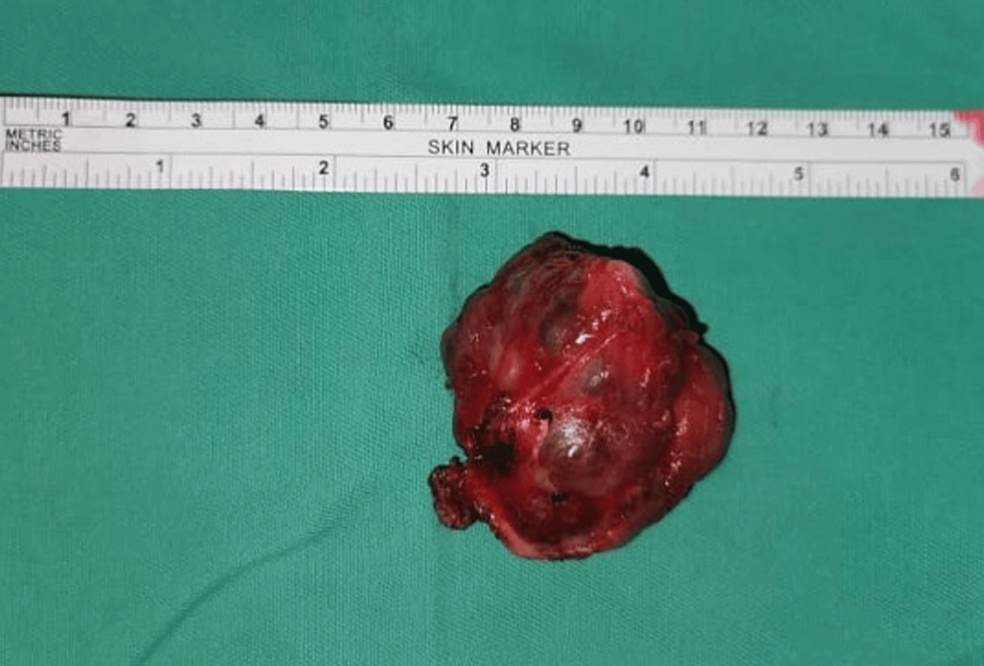
Image showing the excised tumor, which measured approximately 5 x 4 cm

**Figure 5:**
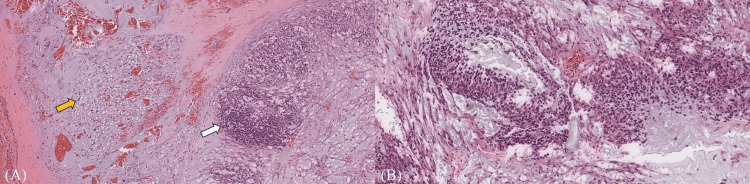
Histopathological image Histopathological image showing (A) stromal components with spindled cells (yellow arrow) and plasmacytoid cells (white arrow). (B) Histopathological image revealing epithelial and myoepithelial components that are consistent with pleomorphic adenoma.

She underwent an excision of the left posterior neck mass under general anesthesia. Intraoperatively, there was a multilobulated tumor with a clear plane with the trapezius muscle (Figure 4). The tumor was successfully excised en bloc. Histopathological examination of the tumor revealed a well-circumscribed encapsulated lesion composed of epithelial, myoepithelial, and stromal components in various patterns that were consistent with pleomorphic adenoma (Figure 5). She was discharged without consequences and there was no evidence of recurrence during her six-month follow-up examination.

## Discussion

Pleomorphic adenoma is a benign salivary gland tumor, which is more common in females, with a peak prevalence in the fourth to sixth decades of life [3]. The tumor is usually located in the major or minor salivary glands while its occurrence in the prevertebral soft tissue is extremely rare. To our knowledge, there is no previous literature that reports on pleomorphic adenoma in the prevertebral soft tissue.

Diagnosing a soft tissue tumor over the head and neck region can be challenging due to its diverse possible histology and complex anatomy of the head and neck region. A recent study conducted in 2021 has identified the common diagnoses for posterior neck masses, which include: lipomas, nuchal fibromas, schwannomas, epidermal inclusion cysts, lipoblastoma, hemangioma, leiomyoma, lymphangioma, and meningioma [4].

In our case, the patient was initially subjected to fine-needle aspiration for cytology (FNAC). It is the investigation of choice in the head and neck due to its high sensitivity (82%) and specificity (95%), cost-effectiveness, and minimal invasiveness [5]. It also carries a minimal risk of potential tumor seeding compared to an open biopsy. However, FNAC cannot determine the histological subtype of the soft tissue tumors for which its success rate in correctly determining the histological subtype ranges from 50-70% [2]. FNAC is also an operator-dependent procedure and the rate of inconclusive results is reported between 3% and 30% [6]. A heterogeneous and large tumor may also be misdiagnosed due to sampling bias, as small amounts of aspirates may not be representative of the tumor.

A core needle biopsy (CNB) was performed, as the initial FNAC result was questionable in the prevertebral soft tissue. CNB has a higher sensitivity and specificity, 96.3% and 99.4%, respectively, especially in differentiating sarcomas from other benign soft tissue tumors [7]. It is also able to differentiate the histological subtypes of the tumor since the tissue samples collected contain a tumor matrix and the tissue architecture is usually preserved. CNB has been shown to have a similar diagnostic value for diagnosing sarcomas and a lower rate of complications compared to incisional biopsy [8]. One of the main complications of performing CNB in the posterior triangle of the neck is iatrogenic spinal accessory nerve injury and the incidence was reported was approximately 3-10% [9].

Radiological imaging provides valuable information in diagnosing soft tissue tumors. A CT scan is a useful tool to diagnose pleomorphic adenoma with a 90% tumor detection rate. The appearance of pleomorphic adenoma in a CT scan is typically featured as a well-defined margin lesion with a smooth border and heterogenous signal on contrast [10]. However, in our case, the tumor had a poor plane with surrounding prevertebral muscles, which prompted us to proceed with an MRI. MRI has superior soft tissue contrast resolution, allowing a better characterization of the internal architecture and the extent of soft tissue tumors [2]. Pleomorphic adenoma is markedly enhanced post-gadolinium administration, and it is possible to detect its capsule in MRI [10]. Therefore, MRI can be one of the options used to evaluate pleomorphic adenoma in the head and neck region [10].

Surgical excision via the transcervical approach is the preferred treatment of pleomorphic adenoma in the head and neck. The tumor has a 6% risk of malignant transformation into carcinoma ex-pleomorphic adenoma or metastasizing benign mixed tumor [11]. Following complete tumor excision, the prognosis of this tumor has been shown to be excellent, with a low recurrence rate of 3% [11].

## Conclusions

Pleomorphic adenoma arising from the posterior soft tissue of the neck is a rare occurrence. Although the diagnosis is possible, multi-modality investigations should be performed to critically evaluate the lesion and provide guidance on acceptable treatment. A surgical excision via the transcervical approach is the preferred treatment with a good prognosis and a low recurrence rate following the complete excision of the tumor.
